# Mm19, a *Mycoplasma meleagridis* Major Surface Nuclease that Is Related to the RE_*Alw*I Superfamily of Endonucleases

**DOI:** 10.1371/journal.pone.0152171

**Published:** 2016-03-24

**Authors:** Elhem Yacoub, Boutheina Ben Abdelmoumen Mardassi

**Affiliations:** Unit of Mycoplasmas, Laboratory of Molecular Microbiology, Vaccinology and Biotechnology Development, Institut Pasteur de Tunis, University of Tunis, El Manar, Tunisia; Institute of Molecular Genetics IMG-CNR, ITALY

## Abstract

*Mycoplasma meleagridis* infection is widespread in turkeys, causing poor growth and feathering, airsacculitis, osteodystrophy, and reduction in hatchability. Like most mycoplasma species, *M*. *meleagridis* is characterized by its inability to synthesize purine and pyrimidine nucleotides *de novo*. Consistent with this intrinsic deficiency, we here report the cloning, expression, and characterization of a *M*. *meleagridis* gene sequence encoding a major surface nuclease, referred to as Mm19. Mm19 consists of a 1941- bp ORF encoding a 646-amino-acid polypeptide with a predicted molecular mass of 74,825 kDa. BLASTP analysis revealed a significant match with the catalytic/dimerization domain of type II restriction enzymes of the RE_*Alw*I superfamily. This finding is consistent with the genomic location of Mm19 sequence, which dispalys characteristics of a typical type II restriction-modification locus. Like intact *M*. *meleagridis* cells, the *E*. *coli-*expressed Mm19 fusion product was found to exhibit a nuclease activity against plasmid DNA, double-stranded DNA, single-stranded DNA, and RNA. The Mm19-associated nuclease activity was consistently enhanced with Mg^2+^ divalent cations, a hallmark of type II restriction enzymes. A rabbit hyperimmune antiserum raised against the bacterially expressed Mm19 strongly reacted with *M*. *meleagridis* intact cells and fully neutralized the surface-bound nuclease activity. Collectively, the results show that *M*. *meleagridis* expresses a strong surface-bound nuclease activity, which is the product of a single gene sequence that is related to the RE_*Alw*I superfamily of endonucleases.

## Introduction

*Mycoplasma meleagridis* is primarily a turkey pathogen, causing poor growth and feathering, airsacculitis, osteodystrophy, and reduction in hatchability [1]. *M*. *meleagridis* can also induce immunosuppression in young birds, thus compromising vaccination programs against other pathogens. Furthermore, *M*. *meleagridis* may cause an inapparent infection which can last for months. During this silent infection, the organism can be harbored by the cloaca and bursa of Fabricius of poults, the oviduct, and by the upper respiratory tract of mature birds [2].

Like all mycoplasmas, *M*. *meleagridis* is a cell wall-less organism with a small genome. The 634,132-bp genome of *M*. *meleagridis* strain ATCC 25294 that we have recently reported [3] is the smallest of the genome sequences hitherto reported for pathogenic avian mycoplasmas [4–7]. The reduced genome of *M*. *meleagridis* makes it particularly deficient in genes controlling several biosynthetic metabolic pathways, such as the oxidative phosphorylation pathway and the tricarboxylic oxidative cycle [8–10].

Among the metabolic pathways that are lacking in the mycoplasmas are those required for pyrimidine and purine biosynthesis [10]. Most mycoplasma species share the inability to synthesize purine and pyrimidine nucleotides *de novo* [11, 12]. Only *Mycoplasma penetrans* has been found to have an orotate-related pathway for converting carbamoyl-phosphate to uridine-5’- monophosphate [13].

The intrinsic deficit in synthesizing nucleotides makes mycoplasmas reliant on their host to provide nucleic acid precursors. The inability of mycoplasmas to synthesize purine and pyrimidine nucleotides *de novo* is reflected in the composition of their minimal growth media. For instance, the high content of nucleic acid precursors in the yeast extract makes it an essential component of mycoplasmas growth media. Supplementing growth media with DNA, RNA, and/or with oligonucleotides has been shown to promote efficient mycoplasma growth [14].

Mycoplasmas have evolved multiple strategies to make nucleic acid precursors available. They encode several transport and salvage enzymes ensuring efficient internalization and recycling of these precursors. Previous studies addressing the ability of mycoplasmas to degrade nucleic acids have revealed nuclease activities associated with the intact cells of several mycoplasma species, including *M*. *meleagridis* [15]. Expression of nucleases by mycoplasmas is likely to be a crucial step to make nucleic acid precursors available for their utilization. Several nucleases have been found to be shared by different mycoplasma species. The gene *mnuA*, encoding a membrane-associated nuclease in *Mycoplasma pulmonis*, has orthologues in *Mycoplasma penetrans*, *Mycoplasma pneumoniae*, *Mycoplasma hyopneumoniae*, *Mycoplasma gallisepticum*, and *Ureaplasma urealyticum* [16, 17]. The nuclease gene *mhp379* of *Mycoplasma hyopneumoniae* has orthologues in *Mycoplasma genitalium* as well as in *Mycoplasma pneumoniae*, *Mycoplasma pulmonis*, *Mycoplasma gallisepticum*, and *Mycoplasma synoviae* [18].

Mycoplasma nucleases display different requirements for divalent cations. The activity of *Mycoplasma penetrans* and *Mycoplasma hyorhinis* nucleases is dependent on Ca^2+^ and Mg^2+^ ions, whereas only Ca^2+^ is required for the *Mycoplasma hyopneumoniae* Mhp379 nuclease [19, 18].

Aside from the crucial role of nucleases in mycoplasma growth and survival, several reports have pointed out their role in pathogenesis [19–23]. Several of these nucleases have been implicated in mycoplasma-mediated host cell cytotoxicity. For instance, the Ca^2+^ and Mg^2+^ ion-dependent endonuclease of *Mycoplasma hyorhinis* induced apoptotic changes in epithelial cells, characterized by internucleosomal degradation of chromatin [22]. A nuclease expressed by *Mycoplasma penetrans* triggered apoptosis in cultured lymphocytes [20]. The apoptotic Mpn133 nuclease of *Mycoplasma pneumoniae*, apart from its important contribution to *M*. *pneumoniae*-associated life cycle events, is implicated in host-associated cytopathologies [24]. Likewise, *Mycoplasma gallisepticum* MGA_0676, a membrane-associated cytotoxic nuclease, induced apoptosis in chicken cells, and is thus considered as an important virulence factor [25].

Although surface nuclease activity has been previously described for *M*. *meleagridis* [15], the identity of the encoding gene sequences has been unknown. In the present study, we identified an open reading frame (ORF) showing characteristics reminiscent of the RE_*Alw*I superfamily of restriction endonucleases that we termed Mm19. We found that the bulk of surface nuclease activity displayed by *M*. *meleagridis* intact cells was associated with the product of Mm19.

## Materials and Methods

### Ethics Statement

This study was approved by the biomedical ethics committee of the Institut Pasteur de Tunis. The immunization of rabbits and collection of sera were carried out in strict accordance with the recommendations in the protocol approved by the biomedical ethics committee of the Institut Pasteur de Tunis (Permit Number: 2015/16/I/LR11IPT01/V0).

### Bacterial Strains, λ Phage, Plasmids and Culture Conditions

*M*. *meleagridis* type strain 17529 (ATCC 25294) was cultured in Frey’s medium [26] supplemented with 15% fetal calf serum. Culture and antigen preparation were performed as described elsewhere [27]. Antigens were prepared from a low-passage stock and were stored at -20°C until they were needed.

*Escherichia coli* strains Y1090r- (Stratagene, La Jolla, Calif.), HB 101 (ATCC 33694) and BL21 (DE3) Star (Promega, Lyon, France) were used for cloning and expression purposes. *E*. *coli* strain Y1090r- was grown in TB medium (1% Bacto Tryptone, 0.5% NaCl, 0.2% maltose, 10 mM MgSO_4_, pH 7.4) at 30°C overnight with shaking. The *E*. *coli* Y1090r- cells were infected with the bacteriophage λgt11 (Stratagene, La Jolla, Calif.) and grown in NZY top agarose on Luria-Bertani (LB) agarose plates containing ampicillin at 100 μg/ml. The *E*. *coli* HB101 and BL21 strains were grown in 2YT medium at 37°C [28].

### Construction and Screening of a *M*. *meleagridis* Genomic Expression Library

An expression library of *M*. *meleagridis* (type strain 17529) genomic DNA was constructed in the λgt11 vector. Immunoscreening and isolation of *M*. *meleagridis*-specific DNA fragments from recombinant λ phages were performed as previously described [29]. In order to favor selection of DNA fragments that encode surface antigens, we screened the *M*. *meleagridis* expression library with a rabbit anti-*M*. *meleagridis* serum that had been depleted of antibodies to the cytosolic proteins. For this purpose, *M*. *meleagridis* culture was sonicated and the supernatant, which contains the bulk of cytosolic proteins, was incubated with the anti-*M*. *meleagridis* serum for 1 h at 37°C. After centrifugation, the serum was incubated for a second round with the cytosolic fraction, clarified, and finally used in immunoscreening. After three plaque purifications, the most reactive λ phage clone (clone 19) was selected.

### Sequencing of Mm19 DNA and Bioinformatics Searches

The DNA fragment in clone Mm19, referred to hereafter as Mm19, was subcloned in plasmid pSPT19 (Amersham Pharmacia Biotech, Buckinghamshire, England) and sequenced on both strands with T7 and SP6 primers, as well as additional internal primers. The nucleotide sequence of Mm19 was generated using the Prism Ready Reaction Dye Deoxy Terminator Cycle Sequencing Kit on an ABI PRISM 377 DNA sequencer (Applied Biosystems). The sequence was edited and analyzed using BioEdit [30]. The full-length ORF sequence of Mm19 was identified using a BLASTN search against the whole genome sequence of *M*. *meleagridis* ATCC 25294 strain (GenBank accession number: JZXN00000000).

### Scanning *Mycoplasma meleagridis* Genome for Nucleases

*In silico* analysis of Mm19 nucleotide and amino acid sequences was achieved by using MolliGen (http://cbi.labri.fr/outils/molligen/) and RAST (http://rast.nmpdr.org/) database tools. A BLAST search [31] was performed using the NCBI web site (http://www.ncbi.nlm.nih.gov/). The MPI bioinformatics tool web site (http://toolkit.tuebingen.mpg.de) was used to perform detailed homology searches (PSI-BLAST) and the InterPro database (http://www.ebi.ac.uk/interpro/) for motif identification. Trans-membrane topography prediction was performed through the PSIPREPD Protein Sequence Analysis Workbench (http://bioinf.cs.ucl.ac.uk/psipred/).

### Expression of Mm19 in *E*. *coli* and Production of a Monospecific Antiserum

Genomic DNA was extracted from *M*. *meleagridis* as described elsewhere [29, 32]. Specific primers; Mm19-BamF (5’-GGCCGGATCCATGTCTAATTCTGTAAAATGG-3’) and Mm19-NotR (5’-GGCCGCGGCCGCTTATTTGAAAATATCGAATAA-3’) were used to amplify the full-length Mm19 coding sequence. The resulting PCR product was excised from the agarose gel, purified with the GFX PCR DNA and Gel Band Purification Kit (GE Healthcare), and digested with *Bam*HI and *Not*I. The resulting Mm19 DNA sequence was subcloned into similarly digested pGEX-4T-1, the prokaryotic GST fusion-based expression vector (Amersham Pharmacia Biotech, Buckinghamshire, England). A recombinant clone, the integrity of which was confirmed by nucleotide sequencing, was introduced into *E*. *coli* strain BL21, and protein expression was induced by addition of 100 mM isopropyl-β-D-thiogalactopyranoside (IPTG). Efficient expression of Mm19 as a GST fusion protein (GST-Mm19) was checked by SDS-PAGE. The GST-Mm19 fusion protein proved soluble and was readily purified using glutathione-agarose beads (Amersham Pharmacia Biotech, Buckinghamshire, England). Protein concentrations were estimated using the bicinchoninic acid method (Pierce, Rockford, IL) with bovine serum albumin as a standard. The purified GST-Mm19 fusion protein was first analyzed by SDS-PAGE and its identity verified by Western immunoblotting, using anti-GST serum.

Purified GST-Mm19 protein was used to immunize New Zealand White rabbits by subcutaneous and intramuscular routes. Inoculations were done using 200 μg of purified recombinant fusion protein emulsified in an equal volume of Freund’s complete adjuvant (Difco Laboratories, Detroit, Mich.). Three subsequent immunizations with the same amount of protein in Freund’s incomplete adjuvant (Difco Laboratories, Detroit, Mich.) were given at two week intervals. Serum against GST protein alone was raised in rabbits using the same protocol as for the Mm19 fusion product.

### Filter Colony Blotting

Discs of nitrocellulose membrane (Bio-Rad) were placed on the surface of agar plates containing fresh *M*. *meleagridis* colonies, marked for orientation, removed, and air dried on filter paper. The membrane was then blocked by incubation in 5% (wt/vol) bovine serum albumin (Sigma-Aldrich) in PBS, for 1 hour at room temperature, and then processed for immunostaining with rabbit anti-GST-Mm19, anti-GST, and anti-*M*. *meleagridis* sera, as previously described [33]. Colored imprints of colonies on nitrocellulose membrane were observed under a microscope at 100 x magnification. To demonstrate that the Mm19 product was surface-exposed, we subjected *M*. *meleagridis* colonies to tryptic digestion on agar plates before their imprinting onto nitrocellulose discs. For this purpose, *M*. *meleagridis* colonies were impregnated with Tris-salt (TS) buffer (50 mM Tris-HCl, 0.145 M NaCl buffer, pH 7.4) containing 500 μg/ml of trypsin (Difco 1:250) for 30 min at 37°C. Trypsin-treated colonies were then washed thrice with TS buffer, imprinted onto nitrocellulose discs, air-dried, and immediately immunostained with anti-GST-Mm19 serum. As a control, colony blotting was also performed in parallel without tryptic digestion.

### Preparation of DNA and RNA Substrates

Closed circular plasmid DNA of pCR2.1 (Invitrogen, Germany) was purified from *E*. *coli* strain Top10 (Invitrogen) with Qiagen Maxi Kit columns (Chatsworth, Calif.) according to the manufacturer’s instructions. Double-stranded DNA was prepared from mycoplasma-free Vero cells (ATCC CCL-81) using the Easy DNA isolation kit. M13 phage single-stranded DNA was purified with Qiagen Midi kit columns (Chatsworth, Calif.) following the manufacturer’s protocol. Total RNA was purified from exponentially growing *E*. *coli*, strain BL21 (DE3) Star, using the RNeasy RNA purification kit of Qiagen (Chatsworth, Calif.).

### Detection of Nuclease Activity

Nuclease activity and substrate specificity of the GST-Mm19 fusion protein were analyzed by incubating 2.5 μg of the purified protein with 1 μg each of closed circular plasmid DNA (pCR2.1), double-stranded DNA (Vero cells), single-stranded DNA (M13 phage), or total RNA (*E*. *coli* BL21 (DE3) star), in 10 mM Tris buffer (pH 8.5). To study the effect of Mg^2+^ and Ca^2+^ divalent cations, 2 mM each of CaCl_2_ and/or MgCl_2_ were added. The reactions were allowed to proceed at 37°C for different time periods (5, 15, 30, 60 and 120 min). Incubations were terminated by the addition of an equal volume of ice-cold 1 M perchloric acid (PCA): l M NaCl. The reaction products were analyzed by 1% agarose gel electrophoresis and ethidium bromide staining.

Nuclease activity associated with intact *M*. *meleagridis* cells was assessed essentially as previously described [15]. Briefly, a mid-log growth phase of *M*. *meleagridis* culture was centrifuged at 12 000 x *g* for 30 min, gently washed twice with 0.01 M sodium phosphate-0.14 M sodium chloride (pH 7.3) (PBS), and suspended in PBS. Endonuclease activity was assessed by incubating 25 μl of serially diluted suspensions of *M*. *meleagridis* (starting total protein amount of 1.25 μg) with an equal volume of PBS containing 1 μg of closed circular pCR2.1 plasmid DNA for 1 h at 37°C. The substrate specificity of the nuclease activity expressed by intact *M*. *meleagridis* cells was assessed by incubating 25 μl (1.25 μg of proteins) of *M*. *meleagridis* suspension with different substrates.

Neutralization of *M*. *meleagridis-*associated nuclease activity with the antiserum to the GST-Mm19 fusion protein was performed by incubating 0.312 μg of *M*. *meleagridis* cell suspension (the smallest amount that completely degraded 1 μg of plasmid DNA) with differing dilutions of the anti-GST-Mm19 serum (1/10, 1/100, 1/1000, 1/2000 and 1/4000), for 1 h at 37°C. Then, 1 μg of pCR2.1 plasmid DNA was added and the mixture was incubated for 1 h at 37°C. The reaction products were analyzed as described above.

## Results

### Isolation and Characterization of Mm19, a *M*. *meleagridis* Nuclease-Encoding Sequence Homologous to Members of the RE_*Alw*I Superfamily of Endonucleases

Using an antiserum that had been adsorbed to remove antibodies against *M*. *meleagridis* cytoplasmic proteins, we screened a λ phage expression library of the *M*. *meleagridis* genome. Among the reactive clones that were identified was clone Mm19, which had partial sequence similarity to members of the RE_*Alw*I superfamily of endonucleases. The full-length sequence of Mm19 (MMELEA_03460 mnemonic in MolliGen database) was extracted from the genome sequence of *M*. *meleagridis* that we had recently determined [3]. The full-length gene sequence was PCR amplified and the nucleotide sequence was further confirmed by sequencing (data not shown).

**Fig 1:**
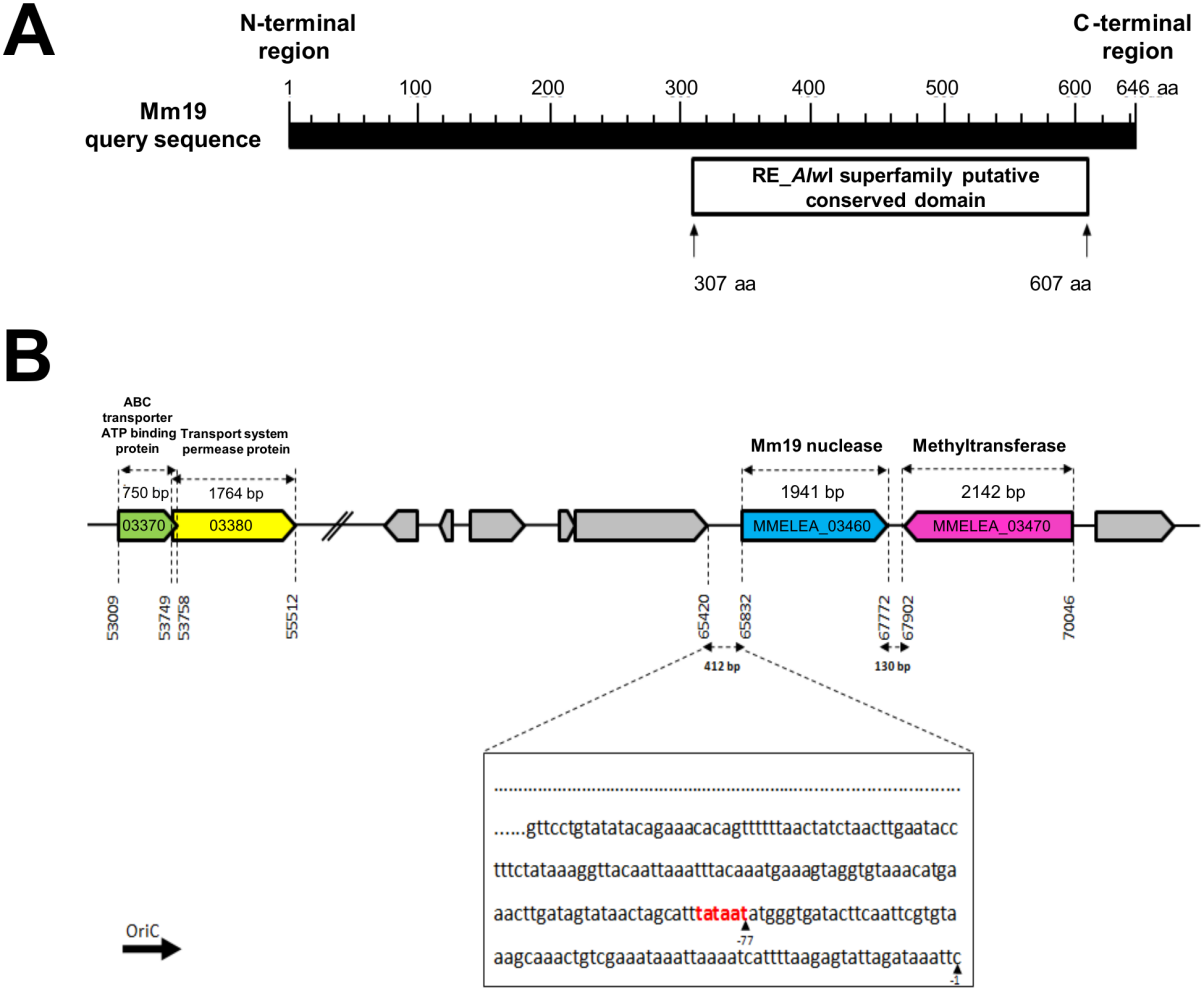
*In silico* characterization of Mm19 nuclease. **(A)** Schematic illustration of RE_*Alw*I conserved domain as determined by BLASTP analysis of Mm19 amino acid sequence. The deduced 300-residue sequence of the RE_*Alw*I superfamily domain mapped to the C-terminal region of Mm19, from residues 307 to 607. **(B)** Genomic location of *M*. *meleagridis Mm19* nuclease ORF and *methyltransferase* sequences. The two genes encoding these enzymes are located next to each other with an intergenic region of 130 bp and opposing orientations; *Mm19* is on the positive strand, while the *methyltransferase* gene is encoded on the negative strand. A “TATAAT box” (in red) was detected upstream of Mm19 ORF, at position -77. In the same orientation and approximately 10 kb upstream of the *Mm19* gene sequence, genes coding for an ABC transporter ATP-binding protein and a transport system permease protein were detected. Genomic coordinates of coding sequences are indicated. All genomic data were extracted from the MolliGen database.

The Mm19 ORF consists of a 1941-bp sequence (GenBank accession number KJ736821), encoding a 646-amino-acid polypeptide with a predicted molecular mass of 74.825 kDa and a theoretical *pI* of about 5.64. The Mm19 ORF contained no opal (TGA) stop codons, known to encode tryptophan in mycoplasmas. A BLASTN search did not detect any significant similarity with any other nucleotide sequence deposited in the GenBank. In contrast, BLASTP/PSI-BLAST analyses revealed a putative conserved domain (pfam09491) related to the RE_*Alw*I superfamily of type II restriction endonucleases (Fig 1A). This domain, which spaned amino acid residue positions 307 to 607, was also detected in proteins of unrelated bacteria and mycoplasmas other than those associated with the avian species (S1 Fig). These include proteins from *Lactococcus garvieae* (59%), *Streptococcus parauberis* and *Streptococcus parasanguinis* (57% and 56% identity, respectively), *M*. *bovis* PG45 and *M*. *mycoides subsp*. *capri* GM12 (49% identity, both), *M*. *agalactiae* (48%), *Streptococcus mutans* (45%), *Veillonella sp*. *HPA0037* (46%) and *Streptococcus pneumoniae* (45%).

### The Sequence Environment of Mm19 Is Typical of a Type II Restriction-Modification System

Examination of *M*. *meleagridis* genome sequence surrounding the Mm19 ORF (MMELEA_03460) revealed a *methyltransferase-*encoding sequence (MMELEA_03470) that is located immediately downstream of it, but in the opposite orientation relative to each other. The intergenic region was about 130 bp (Fig 1B). Such a gene configuration, a *methyltransferase* along with a gene sequence encoding a type II restriction endonuclease, is typical of a type II restriction-modification system. The 412-bp sequence upstream of Mm19 was AT rich (73.5%) and most likely represents the promoter region, inasmuch as a typical “TATAAT box” could be identified at -77 bp from the ATG initiation codon. Moreover, two genes, MMELEA_03370 and MMELEA_03380, that respectively encode an ABC transporter ATP-binding protein and a transport system permease protein, were detected in the positive DNA strand (Fig 1B). In analogy with previously studied nucleases in other mycoplasmas [18, 34], we attempted to identify a transport system that could be associated with Mm19 nulcease. MMELEA_03370 and MMELEA_03380 were found to be the closest genes to Mm19 (MMELEA_03460) that encode ATP-binding cassette (ABC) transporter system components. MMELEA_03370 and MMELEA_03380 are located approximately 10 kb upstream of Mm19, overlapped by 9 bp, and are similarly oriented (Fig 1B). PSI-BLAST searches identified a conserved protein domain (ABC domain of the binding protein-dependent phosphonate transport system) in MMELEA_03370 sequence, which belongs to the ABC_Phnc_Transporter family (aa residues 8 to 244) (S2A Fig). In MMELEA_03380, two conserved protein domains were identified: TM_PBP2, a transmembrane domain subunit found in periplasmic binding proteins (aa residues 98–276), and PhnE, a permease component of ABC-type phosphate/phosphonate transport systems (aa residues 337 to 571) (S2B Fig). Therefore, it seems likely that the encoded products of MMELEA_03370 and MMELEA_03380 form together a phosphonate ABC transport system. Moreover, homologues of both MMELEA_03370 and MMELEA_03380 were identified in many other mycoplasma species and unrelated bacteria, thus suggesting that this ABC transport system is conserved (data not shown).

### Mm19 Is Expressed and Is Surface-Exposed in *Mycoplasma meleagridis* Intact Cells

To confirm that Mm19 was expressed in *M*. *meleagridis* and to characterize its product, we generated an antiserum against its predicted ORF. For this purpose, we expressed the Mm19 full-length sequence in *E*. *coli* as a GST fusion protein. The recombinant product had the expected molecular mass of 104 kDa in SDS-PAGE (S3 Fig, panel A), proved soluble and was successfully purified by affinity chromatography (S3 Fig, panel B). The bacterially expressed GST-Mm19 product was then used to generate a monospecific antiserum in rabbits.

**Fig 2:**
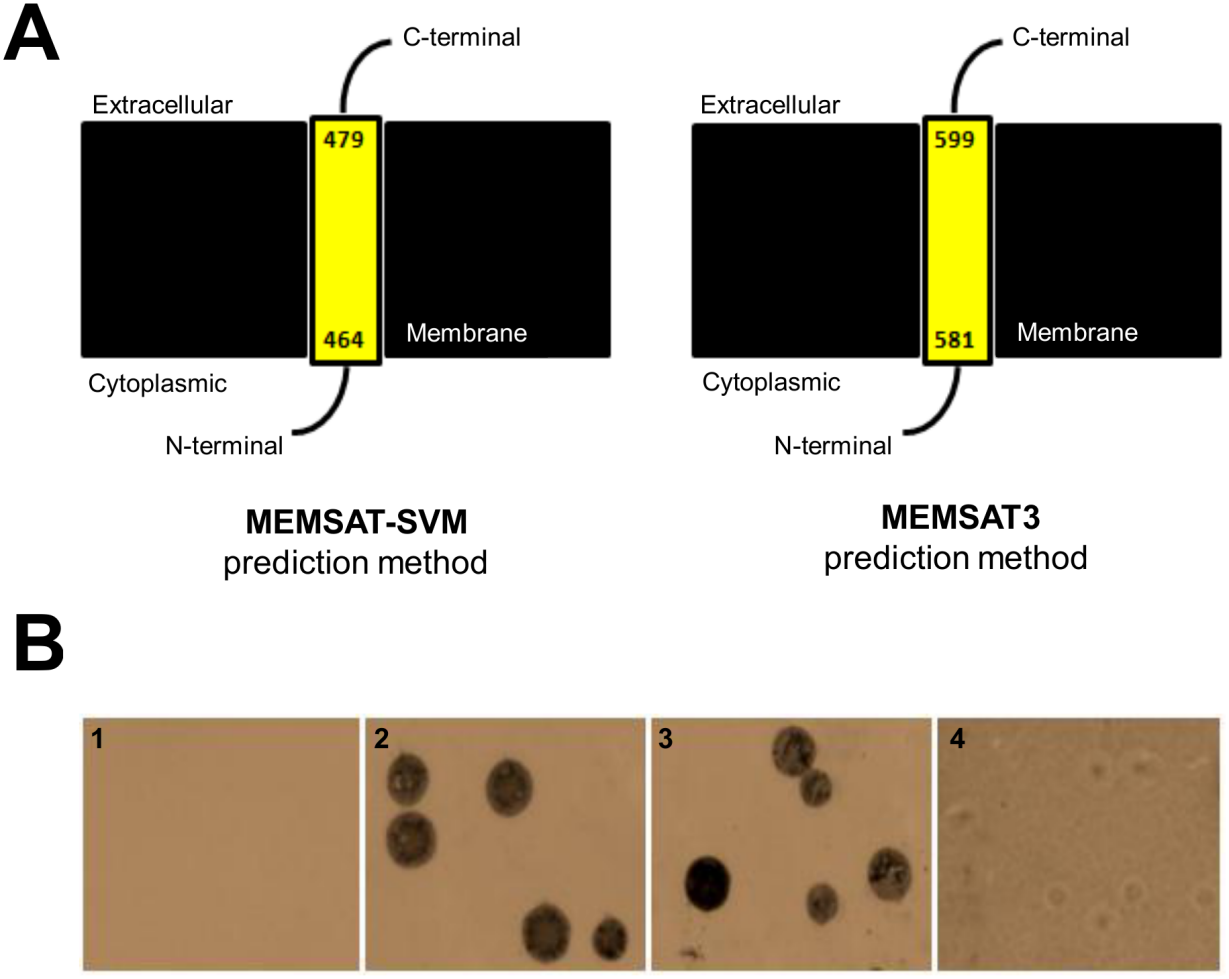
Hypothetical and experimental evidence of Mm19 surface exposure. **(A)** Schematic representation of the secondary structure of Mm19 protein as predicted by PSIPRED based on PSI-BLAST. Using MEMSAT-SVM, a segment of 464 amino acids, including the N-terminal region of the Mm19 nuclease protein, was predicted to be located in the cytoplasm. The C-terminal 167 residues were predicted to be exposed to the extracellular milieu, while 15 amino acid residues were embedded within the membrane. A similar topology was predicted by MEMSAT3 but with a few differences in the segments lengths (cytoplasmic segment, 581 aa; transmembrane segment, 18 aa; extracellular segment, 47 aa). **(B)** Surface expression of *M*. *meleagridis* Mm19 as revealed by colony blotting. *M*. *meleagridis* colonies growing on agar plates were left untreated or subjected to trypsin digestion then imprinted onto nitrocellulose discs. The anti-GST (panel 1) and the specific polyclonal anti-*M*. *meleagridis* (panel 2) sera were used as negative and positive controls, respectively. The reactivity of anti-GST-Mm19 serum against non- and trypsin-treated *M*. *meleagridis* colonies is shown in panels 3 and 4, respectively. Colored imprints of colonies were observed under a microscope at 100 x magnification.

PSIPRED analysis predicted a surface topography for Mm19 product, with N-terminal and C-terminal ends being intracellular and extracellular, respectively (Fig 2A). To confirm the predicted surface location of Mm19 product, we performed colony immunostaining of intact *M*. *meleagridis* cells using the rabbit anti-GST-Mm19 serum. As shown in Fig 2B (panel 3), *M*. *meleagridis* colonies imprinted onto nitrocellulose discs strongly reacted with the anti-GST-Mm19 serum. However, the latter serum did not react with *M*. *meleagridis* colonies that had been treated with trypsin before being imprinted onto nitrocellulose (Fig 2B, panel 4), thus confirming that Mm19 product is surface-exposed.

### Mm19 Protein Exhibits Nuclease Activities against Diverse Nucleic Acid Substrates

**Fig 3:**
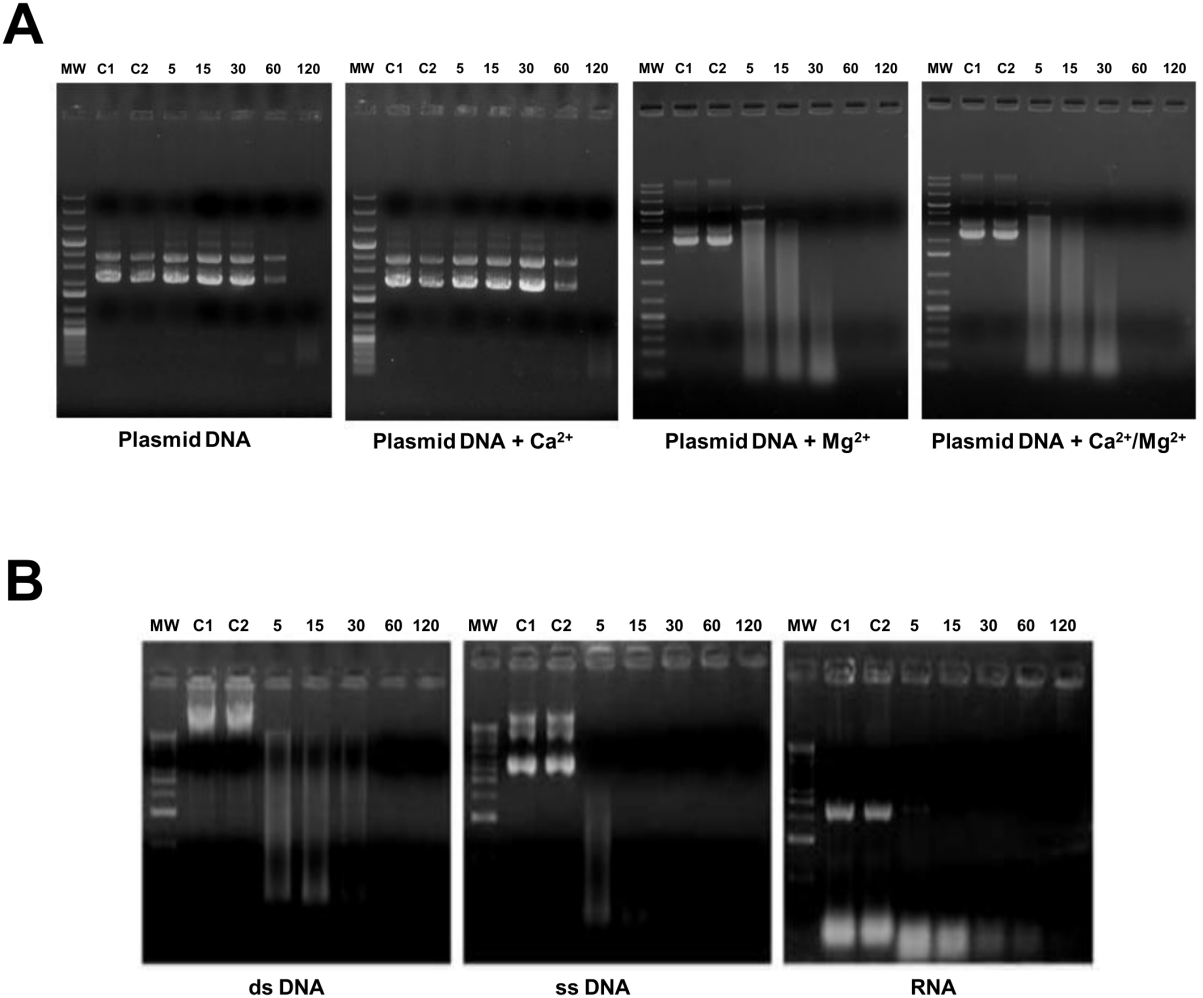
Nuclease activity of GST-Mm19 fusion protein. **(A)** Effect of divalent cations on nuclease activity of purified GST-Mm19 against pasmid pCR2.1 DNA. Nuclease activity was analyzed in the absence or presence of 2 mM of different divalent cations (indicated under each panel). Molecular weight markers, 1 kb DNA Plus Ladder (Thermoscientific), are shown on the left margin of each panel. Lanes C1 and C2 in each panel are plasmid DNA untreated or treated with GST protein, respectively. Purified GST-Mm19 fusion protein was incubated with pasmid pCR2.1 DNA. Incubation times in minutes are indicated above each lane. All reactions were performed at 37°C. **(B)** Substrate specificity of nuclease activity of purified GST-Mm19. Nuclease activity of GST-Mm19 was assessed against Vero cell double-stranded DNA (left panel), M13 phage single-stranded DNA (middle panel), and *E*. *coli* strain BL21 total RNA (right panel). The positions of 1 kb DNA ladder markers are indicated as MW on the left of each panel. Lanes C1 and C2 in each panel indicate end point reactions of the undigested nucleic acid controls after incubation in the nuclease buffer alone or supplemented with GST protein, respectively. Reactions were stopped at the times indicated above each lane.

The finding that the *M*. *meleagridis* Mm19 sequence had significant similarity to restriction endonucleases of the RE_*Alw*I superfamily has prompted us to explore its nuclease activity. First, we assessed the nuclease activity of the recombinant GST-Mm19 against closed circular plasmid DNA. As shown in Fig 3A, DNA digestion could take place in the absence of divalent cations, but was not complete for 2 h (Fig 3A). Addition of Ca^2+^ cations did not significantly enhance DNA digestion, even at concentrations as high as 20 mM (data not shown). In contrast, in the presence of Mg^2+^ cations, plasmid DNA was fully degraded, within 5 min (Fig 3A). Under these latter conditions, all three plasmid DNA forms (supercoiled, linear, and nicked) were digested completely within 1 h. Addition of Ca^2+^ to Mg^2+^ did not enhance the activity (Fig 3A). No nuclease activity was observed when the substrates were incubated with purified GST protein, thus confirming that the observed nuclease activity was due to the Mm19 sequence. Next, we assessed Mm19 nuclease activity against other nucleic acid substrates in the presence of both Mg^2+^ and Ca^2+^. Single-stranded DNA was fully digested after an incubation time of 15 min, which was significantly more rapid than when genomic double-stranded DNA and RNA substrates were used, where complete digestion took about 1 h and 2 h, respectively (Fig 3B).

Collectively the data indicated that the Mm19 product exhibits endonuclease, exonuclease, and RNase activities. The nuclease activity appeared to be more effective against single stranded nucleic acid substrates.

### Intact *M*. *meleagridis* Displays Surface Nuclease Activity Similar to that of Mm19

**Fig 4:**
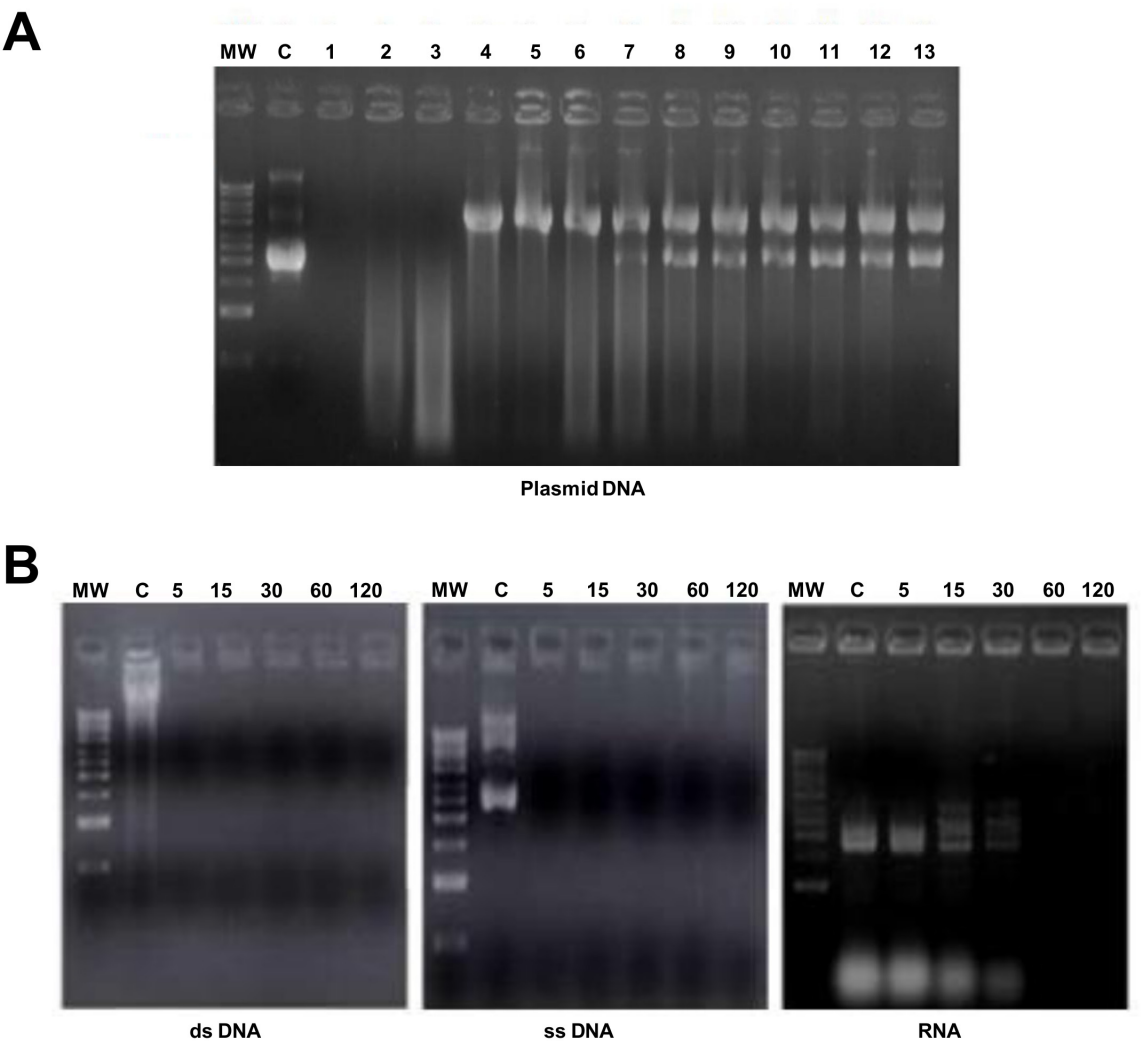
Nuclease activity of *Mycoplasma meleagridis*. **(A)** Nuclease activity associated with intact *M*. *meleagridis* cell suspensions against plasmid pCR2.1 DNA. Lane MW, 1 kb DNA ladder (Amersham); lane C, untreated plasmid DNA control; lanes 1–13, plasmid pCR2.1 DNA incubated with two-fold serial dilutions of *M*. *meleagridis* cells, containing 1.25 μg to 0.0003 μg of total cell protein. **(B)** Nuclease activity of *M*. *meleagridis* against various nucleic acid substrates. The three panels from the left to the right show the effect of *M*. *meleagridis* cells on Vero cell double-stranded DNA, M13 phage single-stranded DNA, and *E*. *coli* strain BL21 total RNA. Molecular weight markers (1 kb DNA ladder, Amersham) are shown on the left of each panel. Lane C was loaded with undigested nucleic acid (incubated with buffer alone). *M*. *meleagridis* cell suspensions (containing 1.25 μg total cell protein) were incubated with nucleic acid substrates at 37°C for different period times (indicated in minutes above each lane).

To confirm previous studies and to characterize further the reported surface-bound nuclease activity in *M*. *meleagridis*, we exposed different nucleic acid substrates to intact *M*. *meleagridis* cells. As shown in Fig 4, 1 μg of closed plasmid DNA was degraded completely within 1 h in presence of intact *M*. *meleagridis* cells (equivalent to 1.25 μg of total proteins) (Fig 4A, lane 1). Degradation of plasmid DNA decreased significantly with increased dilutions of *M*. *meleagridis* antigen, as witnessed by the gradual disappearance of DNA smears in the agarose gel (Fig 4A, lanes 2 and 3). The minimum equivalent amount of *M*. *meleagridis* proteins that completely degraded 1 μg of plasmid DNA was determined to be 0.312 μg. We could demonstrate no nuclease activity when the plasmid DNA was incubated in *M*. *meleagridis*-free buffer or in uninoculated culture medium, indicating that DNA digestion was due to *M*. *meleagridis* antigens, and not caused by a contaminant (data not shown). It is noteworthy that at low concentrations of *M*. *meleagridis* whole cells (equivalent to 0.156 μg—0.039 μg of total proteins), only the supercoiled plasmid DNA form was cleaved (Fig 4A, lanes 4–6), suggesting that *M*. *meleagridis* is endowed with an endonuclease nicking activity against closed circular plasmid DNA. *M*. *meleagridis* intact cells also had strong nuclease activity against double stranded and single stranded DNA substrates, which were digested completely within a 5-min incubation period. However, complete digestion of RNA by *M*. *meleagridis* took a more prolonged incubation time (60 min) (Fig 4B).

### Mm19 Product Accounts for the Bulk of *M*. *meleagridis* Surface Nuclease Activity

**Fig 5:**
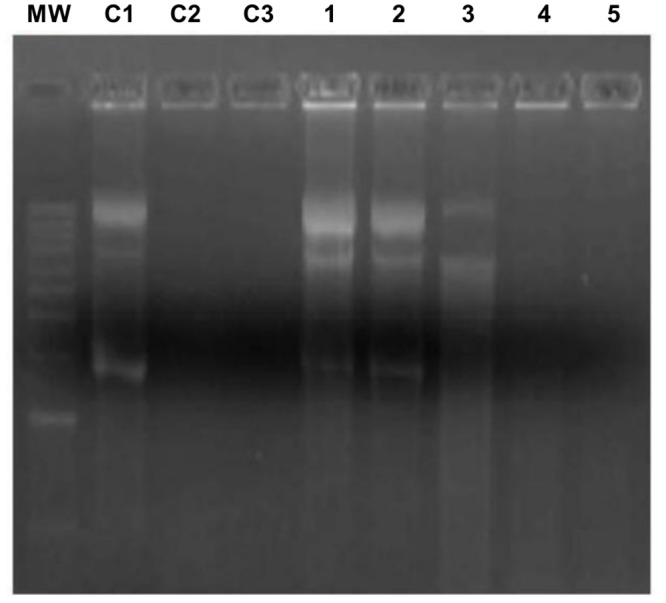
Neutralization of *M*. *meleagridis* membrane-associated endonuclease activity. Nuclease activity neutralization was tested with three controls (lanes C1, C2, and C3). Lane C1, plasmid pCR2.1 DNA incubated in reaction buffer only; Lane C2, plasmid pCR2.1 DNA incubated with untreated *M*. *meleagridis* cells; Lane C3, plasmid pCR2.1 DNA incubated with intact *M*. *meleagridis* cells that had been treated with rabbit antiserum against GST. Lanes 1–5, plasmid pCR2.1 DNA incubated with *M*. *meleagridis* cells treated with rabbit anti-GST-Mm19 antiserum diluted 1/10, 1/100, 1/1000, 1/2000, or 1/4000, respectively. Lane MW, 1 kb DNA ladder (Amersham).

To determine the extent to which Mm19 contributed to the surface nuclease activity of *M*. *meleagridis*, we evaluated the residual nuclease activity of intact *M*. *meleagridis* cells that had been pre-incubated with anti-Mm19 serum. As shown in Fig 5, the antiserum to Mm19 completely inhibited *M*. *meleagridis-*induced plasmid DNA degradation (Fig 5, lanes 1–3). Such inhibition was lost with increased dilutions of the anti-GST-Mm19 serum (Fig 5, lanes 4 and 5). Likewise, the nuclease activity of *M*. *meleagridis* intact cells against double-stranded DNA, single-stranded DNA, and RNA was completely abolished with the anti-Mm19 serum (data not shown). In contrast, no degradation could be observed when nucleic acid substrates were incubated with *M*. *meleagridis* cells that had been incubated with the rabbit pre-immune serum (data not shown) or with the anti-GST serum (Fig 5, lane C3). Also no endogenous nuclease activity could be demonstrated when DNA or RNA molecules were incubated with the rabbit serum to Mm19 (data not shown). Taken together, these results indicate that the bulk of nuclease activity expressed on the surface of *M*. *meleagridis* cells is associated with Mm19.

## Discussion

The minute size of mycoplasma genomes severely limits their biosynthetic capabilities, leading to a dependence on the host for metabolic precursors [35, 36]. Hence, mycoplasmas might be expected to have evolved efficient mechanisms to ensure the acquisition of these essential nutrients, relying mostly on their surface proteins [8, 37, 38].

Here, by focusing on the avian mycoplasma *M*. *meleagridis*, we have identified a gene sequence, referred to as Mm19, encoding a surface-bound protein with nuclease activity. A BLASTP search of the GenBank database revealed significant sequence similarity with members of the RE_*Alw*I superfamily (pfam09491), which includes several type II restriction endonucleases such as *Alw*I, *Bsp*6I and *Bst*NBI. Mm19 had significant similarity with *Alw*I-related sequences in other mycoplasmas (*M*. *bovis*, *M*. *mycoides* subspecies *mycoides* and *M*. *agalactiae)* and other bacterial species, including several *Lactococcus* and *Streptococcus* species. However, a homolog of Mm19 could not be identified in other avian mycoplasma species. The fact that Mm19 was restricted to *M*. *meleagridis* may reflect a specific metabolic requirement, which is in line with its relatively small genome size compared to the other pathogenic avian mycoplasmas [3–7].

Several membrane-associated and/or released nucleases have been described in other mycoplasma species [17, 18, 19, 23, 24, 34]. These nucleases are thought to play a metabolic role in the production of nucleotide substrates from host or microbial nucleic acids, but they are also involved in a variety of other cellular processes [8]. Among the avian mycoplasmas, nuclease activity has been demonstrated in *M*. *gallisepticum* [39], which could be associated with one of the three potential nuclease sequences identified in the genome sequence of strain R [40]. However, we could not detect any significant sequence similarity with *M*. *meleagridis* nuclease Mm19.

Based on sequence alignments, the Mm19 sequence was found to harbor almost the whole DNA binding domain of *Alw*I enzymes and the complete catalytic/dimerization domain [41]. This finding is consistent with our observation of its Mg^2+^-enhanced nuclease activity, which is seen with type II restriction endonucleases [42].

Scrutiny of the sequence environment of Mm19 nuclease identified two gene sequences (MMELEA_03370 and MMELEA_03380) forming a phosphonate ABC transport system that is located 10 kb apart from the Mm19 sequence. In *M*. *synoviae*, ABC transport system genes involved in the uptake and transportation of nucleotide precursors were found to be located 59 kb away from their corresponding nuclease, MS53_0284, a homolog of the *Staphylococcus aureus* SNase nuclease [18, 34]. Therefore, in spite of their remote location, one can rightfully argue that the encoded products of *M*. *meleagridis* MMELEA_03370 and MMELEA_03380 could be responsible for the uptake and transportation of Mm19-derived nucleotide precursors.

Our data suggest that Mm19 is responsible for the bulk of *M*. *meleagridis* surface nuclease activity, previously described by Minion *et al*. [15]. Indeed, intact *M*. *meleagridis* cells displayed the same substrate specificity and ion requirements as Mm19. Furthermore, the antiserum raised against Mm19 strongly reacted with intact *M*. *meleagridis* cells and abrogated almost all its surface-bound nuclease activity. The finding that a single mycoplasma gene product could be responsible for the majority of the cellular nuclease activity detectable *in vitro* is not without precedent. Indeed, in *M*. *bovis*, it has been recently demonstrated that disruption of a membrane nuclease gene (MBOVPG45_0215) greatly reduces both exonuclease and endonuclease cellular activities [43].

Careful inspection of the coding repertoire of the genome of *M*. *meleagridis* revealed the existence of 12 additional potential nuclease gene sequences (S1 Table), two of which, MnuA-DNAse-like and endonuclease I, are predicted to be membrane-associated, as they have typical N-terminal signal peptide cleavage sites. Hence, we cannot rule out the possibility that other nucleases might be expressed on the surface of *M*. *meleagridis*. Should this be the case, these nucleases appear to be very weakly active compared to Mm19.

*M*. *meleagridis*, the genome size of which appears to be the smallest amongst pathogenic avian mycoplasma species, might be expected to suffer increased genetic constraints because of its reduced coding capacity. The finding that Mm19 is surface exposed and responsible for the majority of *M*. *meleagridis* surface endonuclease activity, suggests that it might be expected to be essential. This Mm19-associated nuclease activity is likely to be one of the mechanisms enabling *M*. *meleagridis* to acquire nucleic acids in the form of free bases and/or oligonucleotides. Nervertheless, the pathophysiological role of *M*. *meleagridis* Mm19 nuclease remains to be explored both *in vitro* and *in vivo*.

Thus, in this study we have confirmed that *M*. *meleagridis* has a surface-bound nuclease activity, and we have shown that it is mainly associated with a gene sequence that is related to the RE_*Alw*I superfamily of restriction endonucleases.

## Supporting Information

S1 Fig Multiple alignment of predicted amino acid sequence of *M*. *meleagridis Mm19* with homologous, *Alw*I-related, proteins in other mycoplasma and bacterial species. Numbers on top indicate the position of the amino acid residue. Identical aa regions are shaded in black while similar aa residues are shaded in grey. Dashes (-) indicate gaps in the amino acid sequence alignment. Mm19, M. meleagridis ATCC, Mm19 protein encoding for *Alw*I family type II restriction endonuclease in *Mycoplasma meleagridis* ATCC 25294 reference strain (Sequence ID: ref| WP_052716983.1|). Hypoth protein, Lacto garvieae, hypothetical protein coding an *Alw*I family type II restriction endonuclease in *Lactococcus garvieae* (Sequence ID: ref|WP_042219559.1|). AlwI Strepto parauberis, *Alw*I restriction endonuclease detected in *Streptococcus parauberis* (Sequence ID: ref|WP_003104805.1|). AlwI Strepto parasanguinis, partial sequence of *Alw*I restriction endonuclease detected in *Streptococcus parasanguinis* (Sequence ID: ref|WP_003013817.1|). Type II RM system, M. mycoides, GCATC-recognizing Type II restriction modification system (MmyCIII) endonuclease subunit of *Mycoplasma mycoides subsp*. *capri str*. *GM12* (Sequence ID: ref|WP_02086294.1|). Hypoth protein, M. bovis, hypothetical protein found in *Mycoplasma bovis PG45* defined as *Alw*I family type II restriction endonuclease (Sequence ID: ref|WP_ 013456255.1|). Type II RM system, M. agalactiae, type II restriction modification system, endonuclease subunit detected in *Mycoplasma agalactiae* (Sequence ID: ref|WP_004024024.1|). Hypoth protein, Strepto mutans, hypothetical protein harboring *Alw*I family type II restriction endonuclease specific domain, identified in *Streptococcus mutans* (Sequence ID: ref| WP_002290248.1|). Hypoth protein, Veillonella sp., hypothetical protein encoding for *Alw*I family type II restriction endonuclease in *Veillonella sp*. *HPA0037* (Sequence ID: ref|WP_016476364.1|). AlwI, Strepto pneumoniae, restriction endonuclease *Alw*I detected in *Streptococcus pneumoniae* (Sequence ID: ref|WP_001237274.1|).
(PDF)

S2 Fig PSI-BLAST analysis of phosphonate ABC transport system proteins identified in *Mycoplasma meleagridis*. **(A)** ABC_phnC_transporter conserved domain (cd03256) detected in MMELEA_03370 amino acid sequence. This domain, spanning amino acid residue positions 8 to 244, corresponds to ATP-binding cassette domain of the binding protein-dependent phosphonate transport system. **(B)** On MMELEA_03380 amino acid sequence, two conserved domains were found. The first corresponds to TM_PBP2 (transmembrane domain subunit found in periplasmic binding protein) (aa residues 98 to 276) and the second to PhnE ABC-type phosphate/phosphonate transport system, permease component (aa residues 337 to 571).
(PDF)

S3 Fig SDS-PAGE analysis and Western immunoblot of the expressed recombinant GST-Mm19 protein. **(A)** Purified GST (lane 1) and GST-Mm19 (lane 2) were separated by SDS-PAGE (12%) and stained with Coomassie brilliant blue. **(B)** Rabbit antiserum raised against GST detected bands of approximately 29 kDa and 104 kDa when used to probe Western blots of GST (strip 1) and recombinant GST-Mm19 protein (strip 2). The BenchMark^™^ Pre-stained Protein Ladder (Novex^®^) was used as molecular weight markers (MW).
(PDF)

S1 Table Nucleases predicted in the genome sequence of *Mycoplasma meleagridis* type strain ATCC 25294. (PDF)
