# Supplementary figures and images for: Mm19, a Mycoplasma meleagridis Major Surface Nuclease that Is Related to the RE_AlwI Superfamily of Endonucleases
Source: PLoS One. 2016 Mar 24;11(3):e0152171. doi: 10.1371/journal.pone.0152171 (PMC4807054; doi:10.1371/journal.pone.0152171)

# A

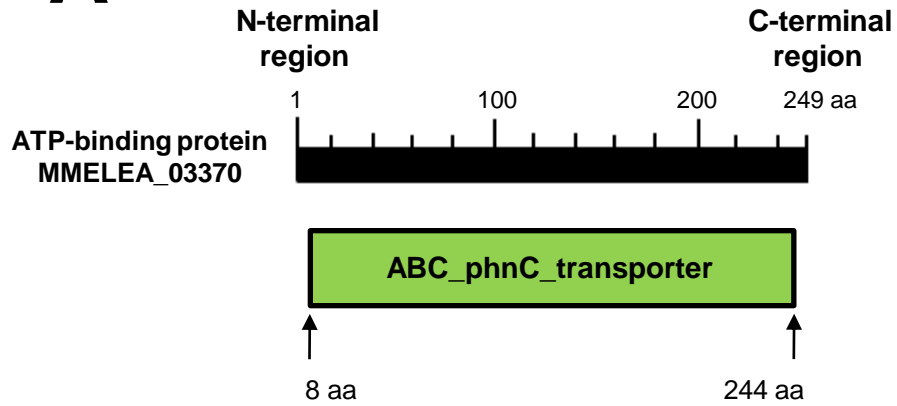

# B

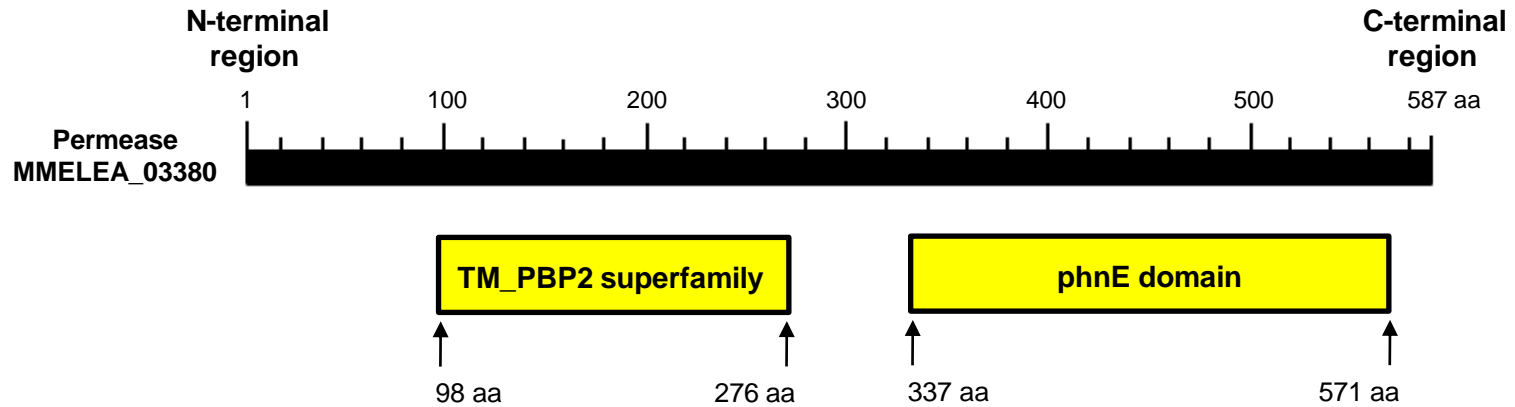

Supplement: S2 Fig — (A) ABC_phnC_transporter conserved domain (cd03256) detected in MMELEA_03370 amino acid sequence. This domain, spanning amino acid residue positions 8 to 244, corresponds to ATP-binding cassette domain of the binding protein-dependent phosphonate transport system. (B) On MMELEA_03380 amino acid sequence, two conserved domains were found. The first corresponds to TM_PBP2 (transmembrane domain subunit found in periplasmic binding protein) (aa residues 98 to 276) and the second to PhnE ABC-type phosphate/phosphonate transport system, permease component (aa residues 337 to 571). (PDF) [file pone.0152171.s002.pdf]

# A

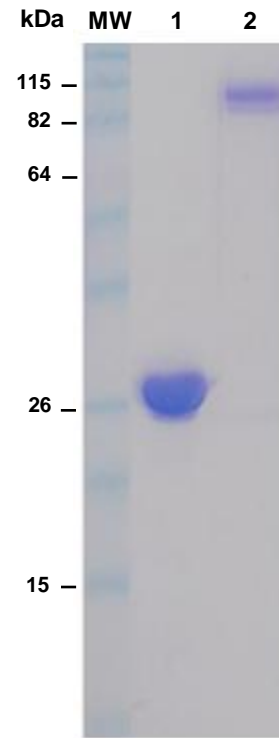

# B

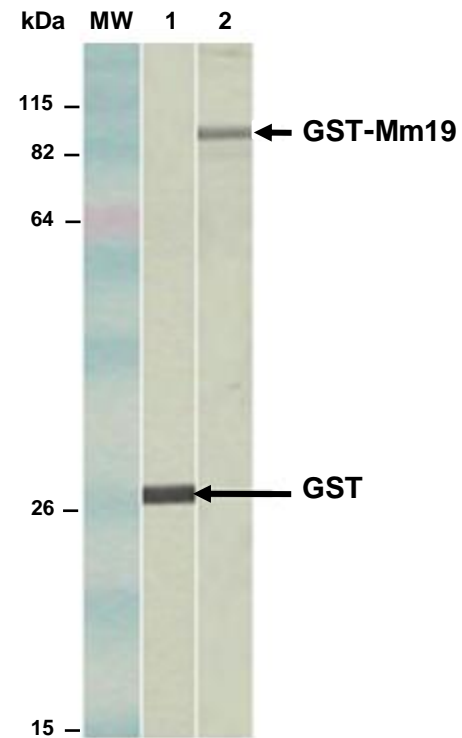

Supplement: S3 Fig — (A) Purified GST (lane 1) and GST-Mm19 (lane 2) were separated by SDS-PAGE (12%) and stained with Coomassie brilliant blue. (B) Rabbit antiserum raised against GST detected bands of approximately 29 kDa and 104 kDa when used to probe Western blots of GST (strip 1) and recombinant GST-Mm19 protein (strip 2). The BenchMark™ Pre-stained Protein Ladder (Novex®) was used as molecular weight markers (MW). (PDF) [file pone.0152171.s003.pdf]
