# Supplementary material for: Mm19, a Mycoplasma meleagridis Major Surface Nuclease that Is Related to the RE_AlwI Superfamily of Endonucleases
Source: PLoS One. 2016 Mar 24;11(3):e0152171. doi: 10.1371/journal.pone.0152171 (PMC4807054; doi:10.1371/journal.pone.0152171)
Supplement: S1 Table — (PDF) [file pone.0152171.s004.pdf]

Table S1. Nucleases predicted in the genome sequence of *Mycoplasma meleagridis* type strain ATCC 25294.

Name	Mnemonic	Strand	Begin	End	Product	Protein size (kDa)	Signal P presence*	Putative conserved domains	
								Name	Position
	MMELEA_00230	-	19801	20493	Ribonuclease HIII	26.6	-	RNase_HII (PF01351)	18 – 228
rnr	MMELEA_00710	+	59569	61716	3'-to-5' exoribonuclease RNase R	82.4	-	OB_RNB (PF08206)	74 - 128
								RNB (PF00773)	245 - 569
								S1 (PF00575)	617 - 693
rnpA	MMELEA_00860	-	78183	78512	Ribonuclease P protein component	13.29	-	Ribonuclease_P (PF00825)	1 - 104
	MMELEA_01340	+	131970	132767	Putative deoxyribonuclease YcfH	30.99	-	TatD_DNase (PF01026)	6 - 259
rnc	MMELEA_01670	+	165195	165884	Ribonuclease III	26.36	-	Ribonuclease_3 (PF00636)	46 - 136
								dsrm (PF00035)	164 - 224
	MMELEA_03220	-	39606	41081	Membrane nuclease	55.98	+	MnuA-Dnase1-like (COG2374)**	150 - 480
	MMELEA_03390	+	55563	57551	Excinuclease ABC subunit B	77.2	-	ResIII (PF04851)	15 - 155
								Helicase_C (PF00271)	469 - 546
								UvrB (PF12344)	553 - 595
								UVR (PF02151)	626 - 660
	MMELEA_03400	+	57594	60431	Excinuclease ABC subunit A	105.62	-	ABC_tran (PF00005)	553 - 595
								ABC_tran (PF00005)	770 - 863
	MMELEA_03460	+	65832	67772	Hypothetical Mm19 nuclease	74.82	-	RE_AlwI (PF09491)	305 - 605
	MMELEA_03600	-	6497	7030	Nuclease	20.84	-	SNase (PF00565)	49 - 168
nfo	MMELEA_04020	+	72849	73679	Endonuclease IV	31.74	-	AP_endonuc_2 (PF01261)	56 - 232
	MMELEA_04950	-	22552	24279	Excinuclease ABC subunit C	67.95	-	GIY-YIG (PF01541)	16 - 91
								UvrC_HhH_N (PF08459)	356 - 504
	MMELEA_05460	+	86764	87960	Endonuclease I	45.11	+	Endonuclease_1 (PF04231)	140 – 373

All reported data are available in Molligen database (http://cbi.labri.fr/outils/molligen/)
Using PSIPRED software, all identified nucleases were found to have a transmembrane localization
*The presence of signal peptide cleavage sites in amino acid sequences of *M. meleagridis* nucleases was screened by SignalP 4.1 server (http://www.cbs.dtu.dk/services/SignalP/)
**This putative domain was detected by submitting amino acid sequence of membrane nuclease of *M. meleagridis* to BLASTP (http://blast.ncbi.nlm.nih.gov/)
