# Supplementary material for: Mm19, a Mycoplasma meleagridis Major Surface Nuclease that Is Related to the RE_AlwI Superfamily of Endonucleases
Source: PLoS One. 2016 Mar 24;11(3):e0152171. doi: 10.1371/journal.pone.0152171 (PMC4807054; doi:10.1371/journal.pone.0152171)
Supplement: S1 Fig — Numbers on top indicate the position of the amino acid residue. Identical aa regions are shaded in black while similar aa residues are shaded in grey. Dashes (-) indicate gaps in the amino acid sequence alignment. Mm19, M. meleagridis ATCC, Mm19 protein encoding for AlwI family type II restriction endonuclease in Mycoplasma meleagridis ATCC 25294 reference strain (Sequence ID: ref| WP_052716983.1|). Hypoth protein, Lacto garvieae, hypothetical protein coding an AlwI family type II restriction endonuclease in Lactococcus garvieae (Sequence ID: ref|WP_042219559.1|). AlwI Strepto parauberis, AlwI restriction endonuclease detected in Streptococcus parauberis (Sequence ID: ref|WP_003104805.1|). AlwI Strepto parasanguinis, partial sequence of AlwI restriction endonuclease detected in Streptococcus parasanguinis (Sequence ID: ref|WP_003013817.1|). Type II RM system, M. mycoides, GCATC-recognizing Type II restriction modification system (MmyCIII) endonuclease subunit of Mycoplasma mycoides subsp. capri str. GM12 (Sequence ID: ref|WP_02086294.1|). Hypoth protein, M. bovis, hypothetical protein found in Mycoplasma bovis PG45 defined as AlwI family type II restriction endonuclease (Sequence ID: ref|WP_ 013456255.1|). Type II RM system, M. agalactiae, type II restriction modification system, endonuclease subunit detected in Mycoplasma agalactiae (Sequence ID: ref|WP_004024024.1|). Hypoth protein, Strepto mutans, hypothetical protein harboring AlwI family type II restriction endonuclease specific domain, identified in Streptococcus mutans (Sequence ID: ref| WP_002290248.1|). Hypoth protein, Veillonella sp., hypothetical protein encoding for AlwI family type II restriction endonuclease in Veillonella sp. HPA0037 (Sequence ID: ref|WP_016476364.1|). AlwI, Strepto pneumoniae, restriction endonuclease AlwI detected in Streptococcus pneumoniae (Sequence ID: ref|WP_001237274.1|). (PDF) [file pone.0152171.s001.pdf]

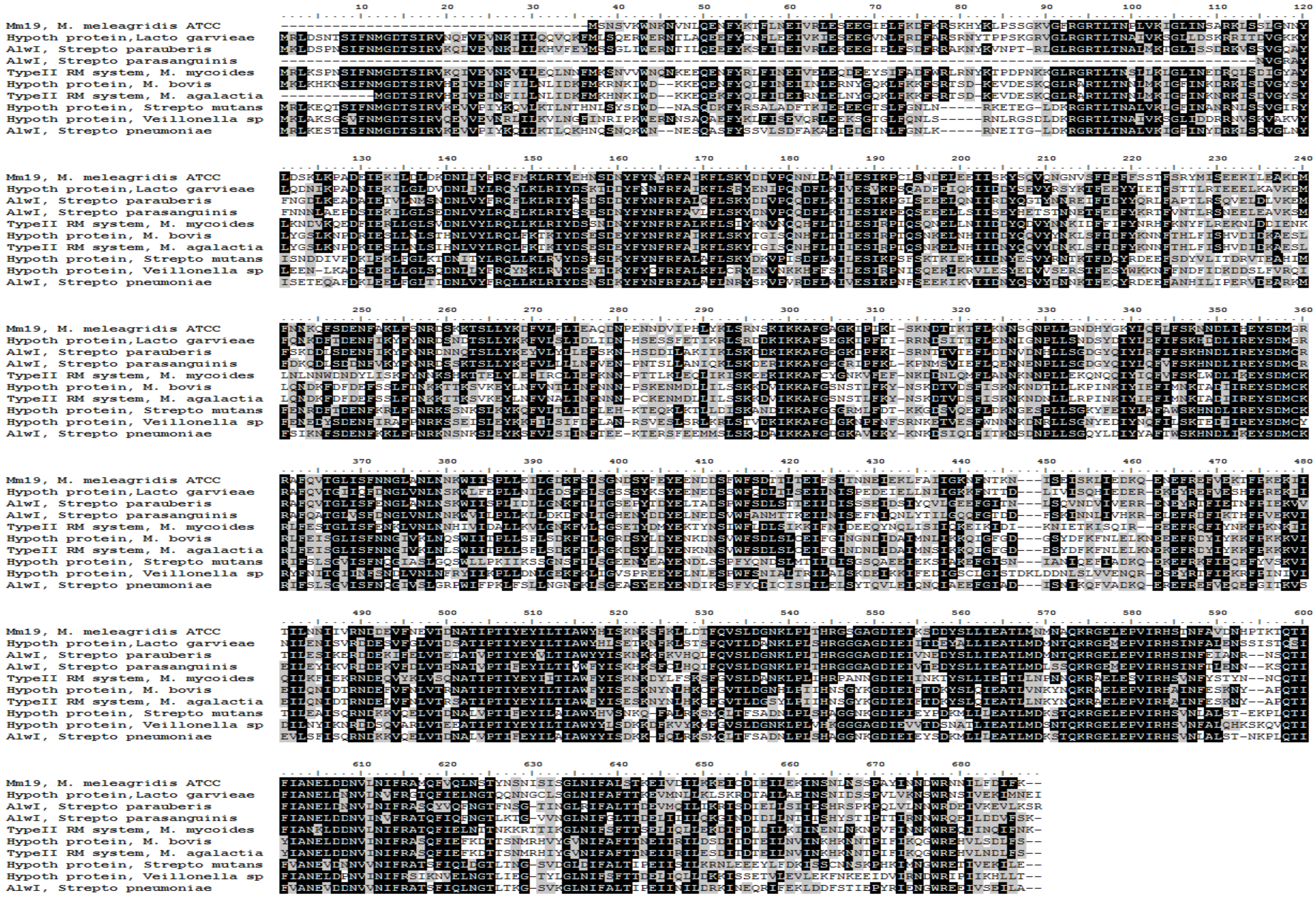

Mm19, M. meleagridis ATCC
Hypoth protein,Lacto garvieae
AlwI, Strepto parauberis
AlwI, Strepto parasanguinis
TypeII RM system, M. mycoides
Hypoth protein, M. bovis
TypeII RM system, M. agalactia
Hypoth protein, Strepto mutans
Hypoth protein, Veillonella sp
AlwI, Strepto pneumoniae
